# Reversible Severe Acute Lactic Acidosis Caused by Thiamine Deficiency in Intensive Care Unit

**DOI:** 10.1155/crcc/3211626

**Published:** 2025-05-08

**Authors:** Jisu Hong, Daehong Cho, Hong Jun Kim, Jaemin Jo, Gil Myeong Seong

**Affiliations:** ^1^Department of Critical Care Nursing, Jeju National University Hospital, Jeju, Republic of Korea; ^2^Department of Internal Medicine, Jeju National University Hospital, Jeju National University College of Medicine, Jeju, Republic of Korea; ^3^Department of Neurology, Jeju National University Hospital, Jeju National University College of Medicine, Jeju, Republic of Korea

**Keywords:** intensive care unit, lactic acidosis, thiamine, Wernicke's encephalopathy

## Abstract

Lactic acidosis is a common cause of metabolic acidosis in hospitalized patients. It is typically caused by hypoperfusion and anaerobic metabolism and is often associated with sepsis. However, it can also result from impaired lactate metabolism, independent of hypoxemia. We report the case of a 50-year-old woman with severe lactic acidosis who was admitted to the intensive care unit. Lactic acidosis was initially attributed to an uncontrolled infection. However, brain magnetic resonance imaging revealed Wernicke's encephalopathy due to thiamine deficiency. The administration of high-dose intravenous thiamine rapidly improved the mental status and normalized serum lactate levels. This case highlights the importance of identifying thiamine deficiency as a reversible cause of lactic acidosis in critically ill patients.

## 1. Introduction

Lactic acidosis is the most common cause of metabolic acidosis in hospitalized patients. In most cases such as those with sepsis, lactic acidosis is associated with hypoperfusion and anaerobic metabolism (Type A lactic acidosis) [1]. Although hyperlactatemia is often attributed to tissue hypoxia, it may result from other mechanisms. For example, Type B lactic acidosis arises from impaired lactate metabolism independent of hypoxemia [2].

Type B lactic acidosis is usually caused by drugs, toxins, hematological malignancies, or nutritional deficiencies. Glycolysis is the metabolic pathway that converts glucose into pyruvate. Pyruvate enters the Krebs cycle for ATP production after being metabolized by pyruvate dehydrogenase into acetyl coenzyme A. Because thiamine serves as an essential cofactor in this metabolic pathway, thiamine deficiency leads to the conversion of pyruvate into lactate [3].

Herein, we describe the case of a 50-year-old woman who developed lactic acidosis while receiving parenteral nutrition.

## 2. Case Report

A 50-year-old woman who had undergone a distal gastrectomy for gastric cancer was admitted to the intensive care unit (ICU) with septic shock. She was unable to progress to a diet because of increased ascites and ileus; therefore, a nasogastric tube was inserted for drainage, and total parenteral nutrition (TPN) was initiated. On ICU Day 1, the patient developed fever and tachycardia and was diagnosed with pneumonia. Broad-spectrum antibiotics and high-dose inotropes were administered to manage septic shock secondary to pneumonia. Her Glasgow Coma Scale (GCS) score was 14. The levels of C-reactive protein and procalcitonin were 15.62 mg/dL and 21.26 ng/mL, respectively. The lactate level was 7.10 mmol/L, measured using the ABL800 FLEX analyzer (Radiometer Medical ApS, Copenhagen, Denmark). However, respiratory distress worsened, leading to the initiation of mechanical ventilation (MV) following endotracheal intubation. By ICU Day 5, as the patient's condition improved, efforts were made to gradually wean her from the ventilator and reduce the use of sedative. However, on ICU Day 7, the patient's GCS score decreased to 8 (E2V2M4), and, despite the discontinuation of the sedatives, no improvement was observed. Additionally, the serum lactate level rapidly increased to 11.43 mmol/L.

**Figure 1:**
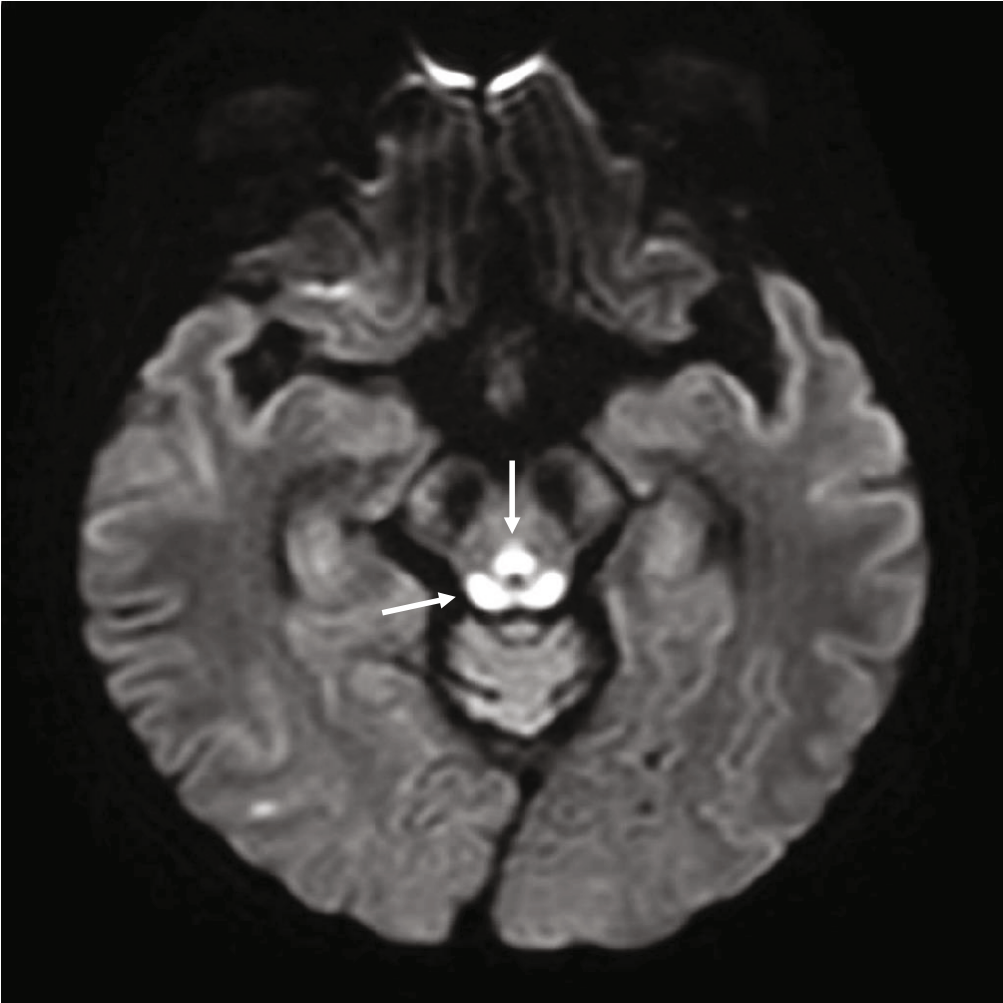
Initial diffusion-weighted image shows the high signal intensities in the pontine tegmentum, periaqueductal gray matter, and tectal plate (arrows).

Initially, the patient's altered mental status was attributed to metabolic encephalopathy associated with sepsis and the use of sedatives for MV. In response to the concern about a hidden infection potentially contributing to her lactic acidosis, the antibiotics were escalated. Despite these interventions and improvements in her overall condition, her mental status did not show corresponding improvement. This led to a neurology consultation to investigate other potential causes of her persistent altered mental status. Due to the patient's decreased level of consciousness, which limited a thorough physical examination, further diagnostic imaging was considered necessary. Brain magnetic resonance imaging (MRI) revealed symmetric changes in the thalamus and brain stem, findings strongly indicative of Wernicke's encephalopathy (WE) (Figure 1).

**Figure 2:**
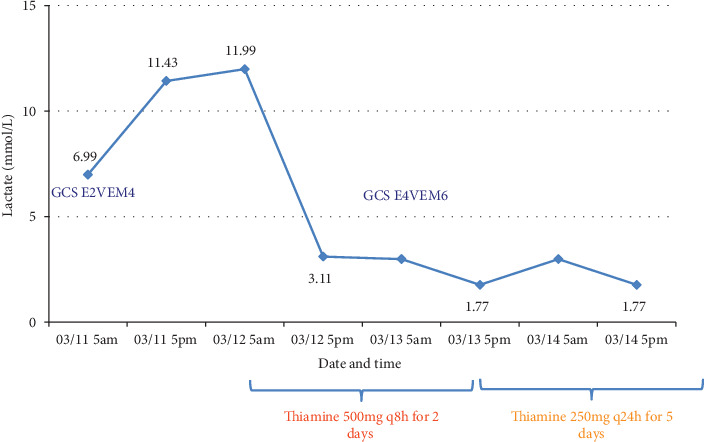
The blood lactate levels after thiamine administration. Following the administration of high-dose thiamine, lactate levels rapidly decreased, and the Glasgow Coma Scale (GCS) improved.

Based on these MRI findings, high-dose thiamine was immediately administered intravenously. Following the administration of thiamine, the patient's consciousness improved, and the blood lactate levels rapidly normalized (Figure 2).

## 3. Discussion

Patients admitted to the ICU often fast for various reasons, which can lead to a range of complications. Fasting in ICU can lead to nutritional deficiencies, and it is important not to overlook that even with TPN, vitamin deficiencies can still occur [4].

Thiamine, a water-soluble vitamin, plays a critical role in energy metabolism and is essential for the growth, development, and function of cells. In glucose metabolism, thiamine acts as a cofactor for pyruvate dehydrogenase [5]. Without sufficient thiamine, pyruvate is unable to enter normal aerobic metabolism. Instead, it is converted to lactate, resulting in the production of only two ATP per glucose molecule and leading to lactate accumulation. Thiamine deficiency is the primary cause of WE, which is characterized by mental confusion, nystagmus, and ataxia, and may lead to high-output cardiac failure [6, 7]. If left untreated, chronic thiamine deficiency can progress from WE to Korsakoff's syndrome, resulting in permanent cognitive deficits such as memory loss and confabulation, as well as polyneuropathy [3, 8].

Type A lactic acidosis is significant in critically ill patients as it can result from conditions like shock, hypoxemia, anemia, and seizures [9]. In contrast, Type B lactic acidosis is rare and often overlooked by clinicians. It typically occurs due to metabolic disorders that affect lactate clearance, such as liver dysfunction, certain medications, malignancies, and inherited metabolic diseases. Thiamine deficiency is another cause that affects lactate metabolism independently of hypoxemia [2]. In the ICU, diagnosing thiamine deficiency may be delayed because clinicians often focus on diagnosing and treating primary diseases, and various causes of altered consciousness are present. Moreover, thiamine deficiency is not commonly considered a cause of lactic acidosis [10]. Critically ill patients are at high risk for thiamine deficiency due to increased metabolic demands and the prevalence of malabsorption or malnutrition [11, 12].

In this patient's case, while factors like sepsis, MV, and the presence of cancer might have contributed to the elevated lactate levels, the lactate level was exceedingly high compared to the general clinical presentation. Considering the patient's history of distal gastrectomy and recent fasting, she was at a very high risk for thiamine deficiency. The MRI findings and the rapid decrease in lactate levels following thiamine administration suggest that thiamine deficiency was likely the primary cause of the lactate elevation.

Decreased consciousness is common in the ICU and directly affects patient outcomes. However, timely and accurate diagnosis is challenging due to the use of various sedatives and the presence of severe underlying conditions. WE is typically diagnosed by the presence of the classic triad of ophthalmoplegia, ataxia, and confusion. However, in ICU patients, these typical manifestations are rare, and unexplained decreased consciousness is often the primary symptom. In such cases, imaging, particularly brain MRI, becomes a vital tool for differentiating the causes of altered mental status. WE is associated with characteristic MRI findings [13]. Typical MRI findings in WE include symmetrical signal intensity changes in the mammillary bodies, medial thalamus, periaqueductal gray matter, and periventricular region. Awareness of these characteristic MRI changes can significantly aid in accurate diagnosis. Early detection and treatment of WE are critical, as the condition is often reversible with prompt thiamine administration. Therefore, understanding the importance of early imaging and recognizing MRI patterns are vital in managing critically ill patients with altered consciousness [14].

In conclusion, we present a case of early diagnosed WE in a patient with septic shock, lactic acidosis, and impaired consciousness. In the ICU, lactic acidosis and impaired consciousness are common findings, but careful consideration of WE as a differential diagnosis is essential. As shown in this case, timely recognition and administration of thiamine can lead to rapid improvement.

## Data Availability

The data supporting the findings of this study are available from the corresponding author upon reasonable request and with appropriate permission.
